# Empirical Phenomenological Inquiry: Guidance in Choosing Between Different Methodologies

**DOI:** 10.1177/23333936231173566

**Published:** 2023-05-17

**Authors:** Joakim Öhlén, Febe Friberg

**Affiliations:** 1 Institute of Health and Care Sciences and Centre for Person-Centred Care (GPCC), and Sahlgrenska University Hospital, Palliative Centre, University of Gothenburg, Gothenburg, Sweden; 2 Faculty of Health Sciences, Stavanger, University of Stavanger, Norway

**Keywords:** hermeneutics, methodology, phenomenological research, phenomenology, research methodology, qualitative methods

## Abstract

Empirical phenomenological inquiry and analyses are of high relevance and applicability for nursing and health care. Phenomenology has clear roots in philosophy, which needs to be brought into an empirical phenomenological inquiry. However, all study of phenomena and experience does not qualify as phenomenological inquiry. The aim of this article is to provide guidance for how to relate different empirical phenomenological methodologies that are in play in the broader field of healthcare research, and thus support healthcare researchers in navigating between these methodologies. For pedagogical purposes, we present commonalities and differences as related to descriptive and interpretive phenomenological inquiries throughout the research process. The merits and criticisms of empirical phenomenological inquiry are commented on.

Historically, empirical research traditions based on phenomenological philosophy have been developed and used across several disciplines and fields of knowledge, including nursing and the broader field of healthcare research (K. Dahlberg et al., 2008; van Manen, 2014). Since 1990 *phenomenological research* is included as a subject heading in the database *Cumulative Index to Nursing and Allied Health Literature* for “research designed to discover and understand the meaning of human life experiences.” The majority of references indexed under this term appear after the millenium shift. Another database, PubMed, includes no specific Mesh-term for phenomenology, but a broad open search with related terms shows increasing occurrence of the term since the millenium shift. These two examples reflect the broad cross-disciplinary interest in experiential and lived experience research. However, compared to this broader field, empirical phenomenological inquiry is a field of methodologies^1^ based on phenomenological philosophy with analysis of phenomena. It is interesting that empirical phenomenological methodological debate was largely reported in the previous century (e.g., Baker et al., 1992; Koch, 1995) but it is following the millenium shift that we recognize methodological studies into specific empirical phenomenological methodologies (e.g., Charalambous et al., 2008; Thomas, 2005). Nevertheless, guidance on how to choose between different empirical phenomenological methodologies is sparse.

Empirical phenomenology inquiry has been regarded as being a spot on match with common challenging research areas in nursing and health care, especially concerning phenomena related to health, illness, suffering and grief. Presentation of empirical phenomenological inquiry is made by using both the concept and the term (i.e., “phenomenological” inquiry/research/method), and also using the conceptual ideas from phenomenology in relation to other methodological umbrella terms.^2^ Nevertheless, some empirical phenomenological inquiries can be criticized for a restrained or insufficient use of the phenomenological underpinnings, as well as for applying the methodology too instrumentally and deviating from the phenomenological purpose. For example, from an analytic philosophical tradition, Paley (2017) argues that references to phenomenological philosophy sometimes (according to him, too often) appear to be included only for rhetoric purposes. Although we do not incline to the same philosophical orientation, we take such critique as a pretext to the need for actually making use of any methodology, explicating and applying assumptions and choices of philosophical nature. For this reason, enhancing rigor is very important if we are to critically elaborate on empirical phenomenological methodologies. Furthermore, empirical phenomenological inquiry employs several specific methodologies characterized by major commonalities, with some differences. This contributes to challenges when designing a study.

The aim of this article is to provide guidance for how to relate different empirical phenomenological methodologies that are in play in the broader field of healthcare research, and thus support healthcare researchers in navigating between these methodologies. In so doing, we primarily intend to support junior researchers considering the choice of empirical phenomenological methodology. The guidance relates to common methodologies used in this field in recent decades. Notably, we have not excluded methodologies that have been critiqued in the literature, for example, for their superficial or vague grounding in phenomenological philosophy. For this reason, we would again like to remind the reader how important it is to read both the original sources for empirical phenomenological methodologies and the original sources for phenomenological philosophy on which they are based, as well as the phenomenological critical inquiries.

Firstly, we elaborate on phenomenological philosophy and what the lifeworld, phenomena and intentionality entail. This is followed by considerations of methodological consequences in which we contrast (in simplified terms) descriptive and interpretive empirical phenomenological methodologies. Even if distinguished, the concepts of description and interpretation are closely related, as argued in debates of both philosophical and empirical phenomenology (P. Ashworth, 2003; K. Dahlberg et al., 2008; Giorgi, 2009; van Manen, 1997). We elaborate on how this is dealt with in the empirical phenomenological inquiry. Then, we explain how different methodologies can be applied to the research process, including formulation of the research problem, sampling, generation of data, analysis and reporting. This is followed by comments on the merits and criticism of empirical phenomenological inquiry and concludes with some reflections about possibilities for phenomenological inquiry.

## Phenomenological Philosophy

Researchers in the field of *phenomenology* usually take their starting point in philosophy, which implies classics are still being read and used, either by reading original philosophical sources or by means of sources specialized in specific classics. The founder of modern phenomenology, Edmund Husserl, who investigated the human mind and what is possible to know about experience, formed a strong critique of reductionism in research (at that time primarily positivism; Husserl, 1970/1900, 1998/1913). His ground-breaking investigations of the directedness of consciousness toward different objects in the world we live in paved the way for a new understanding of human experience. Husserl’s phenomenology was furthered by other philosophers toward a deeper understanding of everyday life and the lifeworld as a basis for human experience. The post Husserlian phenomenologists subsequently both critiqued and gave merit to the heritage of Husserl^3^ and contributed to the tradition of phenomenological philosophy. Among the most notable proponents in this regard are Heidegger (2019/1927), Merleau-Ponty (2014/1945), Gadamer (1989/1960), Ricœur (1984), Schütz (1997/1932) and Sartre (2021/1943). Although differences are discernable in this development, the contribution of these philosophers demonstrates there are certain phenomenological philosophical agreements and continuiuty (Bengtsson, 1991). Here, we briefly return to some basic philosophical phenomenological corner stones which are also of significance in undertaking empirical phenomenological inquiry, namely the lifeworld, phenomena and intentionality.

### The Lifeworld, Phenomena and Intentionality

Husserl brought forward the concept of the *lifeworld* to designate assumptions about both reality (ontology) and knowledge (epistemology). More concretely, the lifeworld is experiential and means “the world in which we are always already living and which furnishes the ground for all cognitive performance and all scientific determination” (Husserl, 1997/1948, p. 41). The lifeworld refers to taken for granted experience, often described as the “the natural attitude.” This is a perceptual and bodily attention to the world we live in. For example, the garden surroundings I walk in (which I am conscious of) are both spontaneous and intuitively experienced even if I hear the birds sing, watch branches move in the wind and sense the stones through my shoes on the gravel path. Husserl (1998/1913), p. 52) puts it as “. . .the free activity of experiencing. . . .” In the words of Zahavi (2019a, p. 145) the natural attitude refers to “the pervasive pre-philosophical assumption that the world can be taken for granted and exists independently of us.” Simultaneously, during the walk I can direct my consciousness to something which draws my cogito out of the natural attitude, the spontaneous and unconscious taken for granted. In empirical phenomenology the interest is, by means of a phenomenological attitude of openness, to enable phenomena which tend to be taken for granted and embedded in the lifeworld to become unpacked and disclosed, and then be analyzed (described and/or interpreted). Collectively, “life” and “world” can be seen as a unity—between subject and object, body and soul, person and others (including the social and societal). However, the link between these dimensions is not complete but ambivalent and tense. Both Husserl and several other philosophers have used and further developed the concept of lifeworld (including the use of varying terminology), so the “lifeworld” does not have one single obvious meaning. Although the ambivalent synthesis between “life” and “world” is a continuum in phenomenological philosophy, differences are notable, for example, as related to the subject being centered (Husserl, 1970/1900, 1998/1913) and de-centered (e.g., Heidegger, 2019/1927), and a clear movement between the centered and de-centered subject (e.g., Merleau-Ponty, 2014/1945). However, within phenomenology the interest is not in the subjective or experiences per se; it is in the relation between an experiencing subject and the object under study (specific phenomenon).

The “lifeworld” brings forward central assumptions about reality; ontological assumptions related to self, body, time, space, relationships and other dimensions. In this way, the philosophy (ontology) will explicate philosophical assumptions which are to be applied in empirical phenomenological inquiry. These ontological assumptions are theoretical by nature but different from, for example, an empirically based theory (as well as from grand theory). Consequently, a feature is that there is no way of escaping the philosophical foundations in an empirical phenomenological inquiry (K. Dahlberg et al., 2008; Giorgi, 2009; van Manen, 1997).

The word phenomenon can be attributed to the Greek word *phainómenon* which means “that which shows itself.” It is of course important to ask: “What shows itself?” The answer will be: “The phenomenon.” In phenomenology any phenomenon is perceived and thus experiential, that is, in the way the object shows itself and appears to someone. For example, my gazing at a flapping bird that suddenly takes off from a tree I passed on my garden walk. I will spontaneously understand this event as something. It makes sense to me in a certain way and is therefore a meaning-making activity. Even aspects that are not perceptually present will be involved in the ways phenomena are understood. Thus, the perceived object is surrounded by other objects. When consciousness is directed to the co-presented, the object will be drawn into a wider contextual understanding. Perceiving a phenomenon happens in a context that is, for example, temporal and spatial. In other words, experience is contextually embedded (Husserl, 1998/1913, p. 52; Schütz, 1997/1932, see also e.g., Bengtsson, 1988; Zahavi, 2019b).

Although the phenomenological understanding of a phenomenon involves a focus on the experiencing person, any phenomena are always assumed to transcend the experiencing person because they are directed toward something other than the self. What the person is perceptually directed at is called “*intentionality*” and this means that our consciousness (and body) is always directed toward something (the phenomenon). What it is directed at may vary, however, as the meaning-making relation is between subject and perceived object. Thus, intentionality is activated in every perceptual act.

The prerequisite for something to appear in perspective is our embodied experience—the body as a subject, the lived body). Building on Husserl’s ideas and critical reading of behaviorist research, the phenomenological notion of embodied experience was furthered Merleau-Ponty (2014/1945). We can never escape the bodily presence. For example, the abrasion from a stone in my shoe makes me sense and notice every step on the gravel path (not only a thought of/cognitive activity but also the unmisstakable bodily sense in the foot). Further, since our experiences are also socially shaped, the lifeworld is at the same time personal and socially shaped and shared. This was furthered by another proponent, Schütz (1997/1932), by means of analyses of everyday situations—in his terms “the everyday lifeworld,” including (and emphasizing) human actions and social practices such as speech and communication.

Hence, in order to undertake empirical phenomenological inquiry, it is important to note the lifeworld refers to what is usually taken for granted, and might be difficult to express in words. At the same time, it is a significant basis of experience from which our thoughts and perceptions are tested and given meaning. Based on ontological assumptions of the “lifeworld,” the taken for granted experiences are both subjective and intersubjective (shared with and shaped in relation to others and objects) and relative. Since the lifeworld is regarded as humanly created and social, it is passed on between people and thus also historical.

## Methodology

In an empirical phenomenological study, the interest is to create as accurately as possible the prerequisites for a phenomenon *to show itself* in ways that enable it to be inquired. More precisely, phenomenological research is about (1) turning to the “things” (the phenomenon) and (2) throughout the inquiry adhering to how “things” are perceived and as a result of the analysis increasing understanding about what a specific phenomenon means (Bengtsson, 2013; K. Dahlberg et al., 2008). Therefore, richness of meaning is a hallmark of both the data and analysis. Of interest is what is directly experienced, as well as the reflected experience of something, such as the experience of being bullied and living with illness.

To illustrate what an inquiry into phenomena entails in a phenomenological sense, the American psychologist Ihde (2012) used a sketch of an ordinary box. To understand what a box is, the person demonstrating must show that the box has a top and bottom, as well as the other sides. If the box is viewed from the front, only one side is visible, but to say that “it is a box,” all sides must be considered and assumed, as well as whether or not it has a lid or an inside. This mindset is akin to what the investigator is looking for in a phenomenological inquiry: to carefully circumscribe and unpack all facets and aspects of the phenomenon. Metaphorically, the phenomenon under study must appear from *all sides*, including much more complicated and difficult-to-define shapes than a simple box. In practice, it is about enabling participants to share as fully as possible and in detail their experiences of the phenomenon being studied; including relating, situating, contextualizing and so forth. When it comes to studying abstract phenomena such as security or violation, it certainly becomes much more difficult to ensure that all “sides” have been included. However, in order for the inquiry to be rich in meaning, openness to a broad range of (i.e., different as well as diverse) experiences of the phenomenon needs to form the data in the study.

Although there are differences in depth and scope, taking starting points in philosophy for the choice of research methodology for a phenomenological inquiry is common across various phenomenological methodologies. The implications of doing so are summarized by Bengtsson (2005) as follows:

- The taken for granted lifeworld must be enabled to become the research object; even though that may be difficult to express verbally, it will be investigated.
- The empirical work is grounded in philosophical assumptions but cannot remain philosophy; the philosophical assumptions (ontological; about reality) must be critically reflected upon and brought into an empirical inquiry.

Phenomenological inquiry has a special significance for conceptualizing how phenomena are experienced and in that sense aims at conceptualization from first-person perspectives (Frank, 1994). Focus is on close-to-data analyses in order to enhance experiential knowledge related to health, illness, suffering and grief, and other lifeworldly shaped phenomena. Even if this is common in other qualitative methodologies, a feature of empirical phenomenological methodologies is the deliberate use of philosophical phenomenology (especially assumptions related to lifeworld, phenomenon, intentionality, etc.) to guide the research process and generate practice-relevant credible knowledge. Interest in phenomenological inquiry can also include providing knowledge for informing practice, such as health care (e.g., H. Dahlberg et al., 2019; van Manen, 2014). In practice fields such as nursing, health care and social work—often impregnated by norms—the phenomenological principle to *turn toward things* may have a special value and may serve as an alternative and complement to, for example, norm-critical analyses. Another aspect of openness is how to handle the researchers’ previous experience, knowledge and understanding of the phenomenon and field under study. For this reason, practices for avoiding unreflected contamination of the research with the researchers’ views and knowledge are emphasized as part of phenomenological methodology (this will be explained as bracketing, bridling and reflexivity under “the research process”).

We notice differing standpoints in the literature in regard to empirical phenomenological methodologies (primarily in textbooks) building on phenomenological philosophy concerning the way this philosophy is related to hermeneutics. Following Palmer (1969), this relates to the extent the lines of phenomenology and hermeneutics are considered to converge in the era of the Heidegger’s philosophy. Since then, some philosopers adopting a hermeneutic stance claim that phenomenology has become more hermeneutic and hermeneutics has become more phenomenological (Bengtsson, 2013; van Manen, 1997). Others claim phenomenology to be a field in itself, with an independent integrity (Giorgi, 2009; Zahavi, 2019a). Still another argument is that ideas such as interpretation are already inherent in Husserl’s phenomenological philosophy (Heidegger, 2019). Here, we refer to phenomenology as a field of philosophy developed over time, including empirical methodological directions.

### Descriptive and Interpretive Phenomenological Methodologies

There are several variations on empirical phenomenological inquiry. In simple terms, these variations can be related to preliminary descriptive and interpretive phenomenological methodologies, as shown in Table 1. However, this distinction requires some initial comments. Those arguing for the use of descriptive analysis avoid steps of interpretion, and in Giorgi’s (2009) terms, this serves as true to data. However, this does not contradict the philosophical assumption (the ontological level) that the human being makes use of interpretation in all meaning-making activites in life. As argued by Merleau-Ponty (2014/1945), the human being is condemned to meaning. However, in a descriptive phenomenological study interpretation is not used as part of analysis. On the other hand, those working with interpretive empirical phenomenological methodologies argue that it is impossible to avoid interpretation as part of an analysis. This does not contradict the inclusion of description in the analysis as a basis for interpretion. Moreover, in interpretive empirical phenomenology, the use of external theory could be included to display layers of meaning as part of an analysis or when it is completed. Others suggest playing down the strict borders between descriptive and interpretive methodologies (H. Dahlberg & Dahlberg, 2020). Although Table 1 is simplified, we believe that the distinction between descriptive and interpretive methodologies serves a pedagogical purpose: to facilitate learning about empirical phenomenological inquiry and how it can be applied overall, and thus not to debate standpoints concerning possible convergences between hermeneutics and phenomenology per se. For in-depth understanding, we recommend reading original sources (references found in Table 1).

**Table 1.** Examples of Phenomenological Methodological Variations With Primarily Descriptive and Interpretive Methods.

Primarily descriptive phenomenological methods	Primarily interpretive phenomenological methods
**Descriptive analysis in search of essence** according to the psychologist Amedeo Giorgi (2009). This empirical method is based on primarily Husserl’s (1998/1913) philosophical phenomenology. Analysis close to the data (mainly interview data) with a focus on searching for and describing the essence of the phenomenon, that is, the invariant meaning that characterizes the phenomenon. A characteristic is to make repeated descriptive condensations of the data. Emphasis is put on the importance of bracketing the preunderstanding and not formulating interpretations during the analysis. ***Empirical Examples*** according to Giorgi are presented in Table 2. Examples of steps in the process of inquiry are also given in Table 2.	**Interpretive hermeneutical phenomenological analysis** or the **phenomenology of practice** according to the pedagogue van Manen (1997, 2014).^a^ This empirical method refers to Husserl (1998/1913) but also philosophers such as Heidegger (2019/1927) and Merleau-Ponty (2014/1945). Of special interest is how a phenomenon appears to somebody or what a lived experience is like. The inquiry process focuses on critical reflection on preunderstanding, to analyze by writing and rewriting in several steps so that the participant’s voice (the narrator), situation and context comes to the fore. The result is a coherent text, rich in meaning, where the phenomenon is interpreted. ***Empirical Examples*** Adams and van Manen’s (2017) study of teaching through phenomenological writing.
**Descriptive phenomenological method** according to psychologist Colaizzi (1978) emphasizes the importance of analyzing the existential meaning in the phenomenon inquired. The analysis is mainly from interview data. These are read to get an overall understanding. Text segments in the data related to the phenomenon (emotions and perceptions) are then marked. From this, meaningful theme clusters (groups of data similar to each other) are created. The intention is to go from “what participants say” to meanings in what is said. The theme cluster is then synthesized to an essential structure. The researcher can return to the participants to validate the results. ***An empirical Example*** of Colaizzi’s phenomenological method is Lam et al. (2020) inquiring a training program intending to support physical activity for children with cancer.	**Lifeworld Phenomenological Research** according to psychologist P. Ashworth (2003, P. D. Ashworth 2006, 2016).^b^ Ashworth’s methodological approach is focused on studying the lifeworld and what that entails for people. He refers to and uses Husserl’s (1998/1913) philosophy, and also Heidegger (2019/1927), Merleau-Ponty (2014/1945), Sartre (1921/1943), and Schütz and Luckmann (1989/1983, 1995/1973). A number of life-world phenomenological concepts are used as analytical tools and to assist the analysis to focus on phenomenological meanings (see Table 3). These concepts or aspects of the life world, or as Ashworth phrased it, fractions, are available in our experiences. By allowing lifeworld aspects to become “glasses” for the researcher, the experience of the phenomenon inquired can get clearer contours in all data. This is of special importance, since lifeworld experiential data is complex and might be embedded and difficult to track. ***Empirical Examples*** according to Ashworth are presented in Table 3, including steps in the process of inquiry. **Interpretive phenomenological approach developed by the nurse researcher**Benner (1994) inspired by Heideggerian (2019/1927) phenomenology. The researcher’s reflection on her own assumptions about what is to be studied is the starting point, often followed by narrative interviews. The data analysis with openness to the preunderstanding is suggested to embrace three interrelated strategies: paradigm cases, exemplars and interpretive thematic analysis. A movement from parts to the whole and back by comparing similarities and differences to identify interpretations and patterns of meanings are suggested. ***An empirical example*** is Massimo et al. (2013) about spouses’ experiences of caring for and living with a partner with Alzheimer’s disease.
**Reflective Lifeworld Research** according to Karin Dahlberg, nursing scholar and colleagues (H. Dahlberg & Dahlberg, 2019b; K. Dahlberg et al., 2008; H. Dahlberg & Dahlberg, 2020). Dahlberg and collegues base their empirical method on Husserl’s phenomenology (1998/1913, but also on proponents such as Gadamer, 1989/1960; Merleau-Ponty, 2014/1945). The method was preliminary developed on the basis of Giorgi’s (2009) descriptive phenomenology. Meaning units are identified in the data analysis and clusters/groups are formed of similar units of meaning . The clusters are then further analyzed in the search for a preliminary understanding of essential meanings. The analysis continues to identify meanings which signify the phenomenon via themes of meanings or “constituents.” This methodology has been further developed with clarification that there is no intrinsic difference between description and interpretation in the analysis (H. Dahlberg & Dahlberg, 2019b, 2020). If an interpretive analysis is made, the descriptive analysis can be clarified by the use of theory and previous research, which is selected after performing the descriptive analysis. The analysis can therefore either focus on describing variations in meanings or describing the invariant essence of the phenomenon. ***Empirical examples*** of the Reflective lifeworld research are Olausson et al.’s (2013) study on the meanings of intensive care patient room as a place of care, and Ozolins et al.’s (2015) study about patients’ experiences in an anthroposophic clinical context.	**A phenomenological hermeneutical method** according to the philosopher, Anders Lindseth and nursing scholar, Astrid Norberg (Lindseth & Norberg, 2004, 2022). The research method is based on the philosophies of Husserl (1998) and Ricœur (1976). The concepts of phenomenon, lifeworld, lived experiences and concrete reflection form the theoretical basis for the method. Data usually consists of narrative interview texts. First a naïve reading is carried out to find an immediate understanding of the text. To validate the naïve reading, a structural thematic analysis is done whereby the text is divided into meaning units, sub themes and themes (eventually main themes).^c^ Finally, a critical interpretation or interpreted whole is formulated whereby the structural analysis is elaborated upon in relation to relevant literature. ***Empirical examples*** Mazaheri et al.’s (2017) study about enrolled nurses’ experiences of good or bad conscience when working with Persian-speaking persons with dementia in a nursing home.
**Additional examples of descriptive phenomenological methods:** **- Phenomenological psychological method** according to the psychologist van Kaam (1969). **- Empirical phenomenological method** according to the psychologist Moustakas (1994).	**Additional examples of interpretive phenomenological methods:** - Empirical lifeworld phenomenology according to the philosopher Bengtsson (1991, 2005, 2013).^d^ See also further exploration of empirical lifeworld phenomenology following the tradition of Bengtsson (I. C. Berndtsson et al., 2007; I. Berndtsson et al., 2019). An empirical example is I. C. Berndtsson’s (2018) study about the use of assistive technology for blind persons based on the concepts of lifeworld and lived body. **- Empirical phenomenological psychological method** (The EPP Method) according to the psychologist Karlsson (1993, 2019) combines phenomenology (see, for example, Husserl, 1998/1913) and hermeneutics (Heidegger, 2019/1927) and other proponents such as Merleau-Ponty, 2014/1945; Schütz, 1997/1932). **- Interpretive phenomenological analysis** (IPA) according to psychologists J. A. Smith et al. (2009) focuses on a disciplinary field of psychology. Underpinnings from phenomenology (Husserl, 1970/1900), hermeneutics (Heidegger, 2019/1927) and ideography (subjective experiences). IPA addresses lived experiences and how to make sense of it in everyday life. For details see J. Smith and Nizza (2022). *Empirical example*: Nizza et al. (2018) study about experiences of pain following participation in a pain management program.

*Note*. Characteristic features are briefly described followed by suggestions with empirical examples. All methods mentioned have been used in different disciplines.

a van Manen’s phenomenological methodology has roots in the Utrecht school of a phenomenological tradition in the Netherlands (Kockelmans, 1987). See also van Manen and van Manen (2021) publication of classic writings from the Utrecht school proponents.

b A nearby methodology to that of Ashworth is the Lifeworld Phenomenological Approach developed by the Swedish philosopher Bengtsson (2005, 2013), see below in Table 1.

c Meaning differences can also be structured in other ways, for example, based on temporal or spatial aspects (Friberg & Öhlen, 2007) and narrative structures (Öhlén et al., 2013).

d Ontological assumptions are in Bengtsson’s (2005, 2013) methodology formulated and actively used in the design of the study, and selected lifeworld phenomenological assumptions are used as guidance in the analysis. To deepen the analysis, additional theories (in addition to the initial lifeworld phenomenological assumptions) are suggested to be incorporated in the interpretations (I. C. Berndtsson et al., 2007).

Although the overall phenomenological foundation is similar, differences in emphasis can be distinguished. Descriptive empirical phenomenological methods are clearly based on Husserl’s philosophical assumptions. Based on detailed scrutinizing of data, the analysis aims to capture the invariant meaning of the phenomenon under inquiry: its essence. In addition to phenomenological philosophy, interpretive empirical phenomenological methods usually also include starting points from philosophy of existence and hermeneutics (Heidegger, 2019). The analysis aims to interpret differences and commonalities in meanings of the studied phenomenon. In this sense, an empirical phenomenological interpretive analysis incorporates a reference to interpretation according to hermeneutic traditions (see Beck, 2021; Chan et al., 2010; Lindseth & Norberg, 2022).

All empirical phenomenological methods have emphasized meanings of phenomena. The goal of the inquiry is similar for all empirical phenomenological methodological variations: to reach understanding guided by the data and as a researcher avoid being captured and remaining in one’s own “natural attitude” and taken for grantedness. Therefore, any phenomenological inquiry involves handling the natural attitude. However, specific strategies for how this can be achieved differ between descriptive and interpretive empirical phenomenological methods:

- Descriptive empirical phenomenological methods emphasize putting the researcher’s own pre-understanding in brackets and carefully distinguishing it from “data” in the analysis. This *bracketing* serves to avoid having the inquiry (all phases of the research process) influenced by the researchers’ own theoretical knowledge, influences, opinions, judgments and personal (usually limited) experiences. H. Dahlberg (2022) refers to bracketing metaphorically in the phenomenological philosophical idea of epoché. An alternative term is “bridling” as suggested by K. Dahlberg et al. (2008); see also H. Dahlberg, 2022) which means a pending and reflective thoughtfulness with openness to different possibilities for what the analysis may reveal.
- Interpretive empirical phenomenological methods emphasize that pre-understanding (including the natural attitude) is a precondition to generating new knowledge and something the investigator has to reflect on, become aware of, and sometimes may need to expand and deepen (e.g., through further fieldwork or using external theory, and reflexivity), and thus make use of. This is necessary for gaining knowledge of meanings embedded in lifeworld phenomena (observed, written and told). Bridling, as described above (H. Dahlberg, 2022), is also applicable in an interpretive empirical methodological study.

Further, descriptive and interpretive phenomenological methods can be related to distinguishing between what constitutes a phenomenon and the fact that it exists, which in turn is about a sometimes assumed idea that phenomenological analysis focuses on the person, intersubjective or lived-experience (or so-called subjective experience, although this concept is rather superficial here). However, based on the idea of the lifeworld, the focus is ultimately a connection between life (the person) and the world (the social). Following this, what constitutes a phenomenon and if it actually exists might be emphasized differently. What constitutes the meaning of, for example, “being offended” can be described and understood without the person feeling violated. Initially, empirical phenomenological analysis (in the spirit of Husserl) sought to analyze what constitutes a particular phenomenon so that its meanings are released from the numerous possible forms of how it exists (different experiences of the phenomenon) in different life situations and circumstances. What constitutes a particular phenomenon becomes aspects of meaning that are invariant and often referred to as the essence, or essence of a phenomenon. From this follows that withholding judgments becomes necessary. This ontological position is usually clear in descriptive methods of empirical phenomenology (see Descriptive Phenomenology Table 1). Notably, withholding judgments is also important in interpretive phenomenological methods but argued for on other bases. Critics (with phenomenological underpinnings e.g., Bengtsson, 2013; I. Berndtsson et al., 2019; see also Paley, 2017) have stated that in having the essence of phenomena as the goal of analysis, there is a risk that there will be no difference between imagining being offended and actually feeling violated. Still, including variations on what the very existence of being offended entails can be crucial in avoiding an idealizing analysis. This ontological position is usually clear in interpretive phenomenological approaches (see Interpretive Phenomenology Table 1). Such latter criticism has been particularly brought forward from existentialist and hermeneutic perspectives (e.g., Chan et al., 2010; van Manen, 2017a).

Some researchers explicitly define themselves by one or the other tradition, while others do not. As mentioned earlier, there are also ongoing discussions as to the problematics of saying an inquiry is either descriptive or interpretive (Beck, 2021; Bengtsson, 1988; Chan et al., 2010; H. Dahlberg & Dahlberg, 2020). Denotations such as *hermeneutical phenomenological* analysis occur (van Manen, 1997, 2014) as well as *phenomenological hermeneutic* analysis (Lindseth & Norberg, 2004, 2022). Again, we want to remind ourselves of the pedagogical purpose of distinguishing between the descriptive and interpretive methods and of the importance of being guided by the original philosophical and methodological sources.

We have outlined below more specifically how empirical phenomenological methods have been applied, using descriptive and interpretive methodologies. In Tables 2 and 3 we use only a few selected references to illustrate this, but the processes of inquiries have broader support in the literature (Beck, 2021; Chan et al., 2010; K. Dahlberg et al., 2008).

**Table 2.** Example of Descriptive Phenomenological Analysis.

Descriptive analysis based on Giorgi and Giorgi (2008, p. 24) and Giorgi (2009, p.128)
1. *Read the data (mostly transcribed interview recordings) to understand the whole without making interpretations of what is read.* 2. *Read all the data again and mark in the text (make a dash with a pen or the like) where the text in any way changes in meaning. Go through the whole material. When this is done, a number of meaning units have been identified.* 3. *Read the identified meaning units again and then, rewrite each meaning unit (transform it) to make it more general and abstract, but still descriptive and close to the data. Often a table is made so that the reader can follow how the transformation of data has been made stepwise. In this way, data is condensed so that what is meaningful in the data segment (that is, meaning unit) becomes unpacked without imposing any interpretation. The purpose is to make visible what the text is about. The condensed meaning units now consist of a text that is shorter than the original data, but they are more at the core.* 4. *Read the condensed meaning units again and determine what constitutes the phenomenon (what characterizes the phenomenon from what the analysis has shown so far). Use that to form what characterizes the phenomenon, that is, its essence.*
***Empirical Examples***: Giorgi and Giorgi’s (2008) study into the phenomenon of learning. Another example is the study by Hemle Jerntorp et al. (2021) about fathers’ lived experiences of caring for their pre-term infant in the neonatal unit and in neonatal home care after the introduction of a parental support program.

**Table 3.** Example of Interpretive Phenomenological Analysis.

Interpretive analysis based on P. Ashworth (2003, P. D. Ashworth 2006, 2016)
1. *Read through all the data to gain understanding of the whole as related to the phenomenon studied.* 2. *Identify and mark text parts that can be related to the following lifeworld aspects: selfhood (interest, choice, priorities, identity), sociality (relationships), body (bodily experience), time, space (experience of space and things, place, rooms and equipment, environment), project (prospects and activities engaged in), discourse (terms, language and expressions) and moodedness (atmosphere and tone in the experience).* 3. *Place all data belonging to one and the same lifeworld aspect together (for example, in a separate file, or use a software for qualitative data analysis) to facilitate overview of the continued analysis. Mark data brought to more than one lifeworld aspect (to keep track). No requirement to find data as related to every lifeworld aspect.* 4. *Read the respective data sets.* 5. *Search and identify the meanings across the lifeworld aspects.* 6. *Identify variations, similarities and differences in meanings. Re-read and examine whether relations between the meanings can be identified, for example, if they highlight different or similar meanings of the phenomenon.* 7. *Write down the meanings of the phenomenon in ways that are rich in meaning—possibly supported by additional theory.*
***Empirical Examples***: P. D. Ashworth (2006) on living with Alzheimer and Friberg and Öhlen (2007) on living with an incurable condition at the end-of-life. See also Andrews et al. (2022).

### Formulation of the Research Problem

Since the lifeworld is complex and differentiated, methodologies are needed that allow generation of data about complexities. This includes people, contexts and the experiencing person, which refers not only to cognitions but also embodiment, sociality and culturality (Bengtsson, 2005). What we are experiencing in life always happens in a lifeworld context. Merleau-Ponty (2014) points out that a person is “condemned to meaning” and thus constantly seeking meaning. In a phenomenological inquiry, the assumption is that life (the person in question) and the world (the context of the experiencing person—the social and contextual in a broad sense) is intertwined.

Research problems where empirical phenomenological inquiry is appropriate usually focus on phenomena that may include multiple meanings, differences in meanings and existential dimensions. Examples are everyday activities, such as having meals, exercising and family gatherings—which in turn may be related to broader phenomena like health, illness, suffering and grief. In an empirical phenomenological inquiry, it is just as appropriate to focus on a particular phenomenon in a more general sense (e.g., crying) as to confine it to certain contexts (e.g., crying in the context of the grief of orphan teenagers who have migrated from a war zone). We would like to stress the importance of delineating the phenomenon of interest as accurately as possible to optimize understanding of what is to be studied and the consequences for the subsequent generation of data.

### Data Generation—Being in the Field

If the phenomenological concept of lifeworld is taken seriously, the point of departure is taken in the life of people in social, bodily, material and cultural contexts. The purpose of the data generation is to create as rich and multifaceted data as possible to give justice to the inquired phenomenon. Thus, how data is created in fieldwork becomes especially important; the researcher(s) needs to become exposed to differences in how the phenomenon under study could be lifeworldly expressed and experienced. This could be achieved through observations, narrative (open) interviews, written narratives or diaries, video or sound recordings and social media, to mention a few possible ways. The richness of the data tends to become more significant than the number of individuals or observations included and will influence credibility of the study. An illustrative example of rich data from fieldwork is displayed in Johansson’s studies (cf. Johansson, 2011; Johansson & Emilson, 2016) of small children in preschool settings. Observations and video recordings of everyday activities were used to disclose ethical values and norms that seemed to guide the children’s interaction in play and communication.

Thus, being in the field means openness to different types of data. For example, in Friberg and Öhlen’s (2007) case study (field work) on learning at the end-of-life, the severely ill person described thoughts and ponderings (narratives) in relation to the ever weaker body and the difficulty of meeting friends because he could not move the way he could before the illness. The researcher participated in settings where the participant was cared for (both out- and in-patient departments in hospitals, as well as hospices) and took part in conversations with healthcare professionals and other activities in the ordinary settings he was involved in. In this case, the generation of data meant following the participant over time and in different settings. In this way, participatory observations were also used as data, that is, situations where the researcher is present and participates in what are ordinary social contexts from the perspectives of the participants. These observations, documented in field notes, were informed by participatory experience or co-created participant experience (Friberg & Öhlén, 2010). Narratives are often used and considered as data in phenomenological inquiries (Polkinghorne, 1995). The philosopher Ricœur (1984) talks about the narrative as a foundational feature in human talk and interactions: storied reasoning. Thus, being human is narrative. Narratives can be defined as having a beginning, a middle and an end, whether it is an everyday story or a life story. Polkinghorne (1995) distinguishes between analysis of narratives (analysis of delimited meaning units to bring out what are common meanings) and narrative analysis (focus on the meaning of the narrative in order to create a new coherent narrative regarding the phenomenon).

A common way of generating data in phenomenological inquiries is via open interviews, dialogical interviews or narrative interviews. There are a number of sources that describe interviewing for the purpose of qualitative research^4^ which are applicable in empirical phenomenological inquiries. In interviews, participants are invited to say what they have experienced, what they have been involved in and how, that is, their lived experiences or experiential perspectives of the specific phenomena. An open introductory question to open up for topics and sharing stories can be posed, for example, “can you tell me what it was like when you understood that your son had hemophilia” (Myrin Westesson et al., 2013). This can be combined with follow-up and probing questions with the purpose of exploring and encircling various possible experiential aspects of the phenomenon in ways that invite the participants to explicate their experience. Such questions are “can you tell me more about this?” or “is there any additional situation you would like to talk about?.”

Regardless of what questions are posed, the asking should involve open questions to find out more that could be related to the phenomenon, while the interviewer is prepared to listen with sensitivity and hold on to something the person just said. Being comfortable with pauses and being silent as an interviewer is often important in giving the participant space to say more without assuming there is necessarily more to say. Disclosing the full agenda right at the beginning of the conversation and doing it in ways that express your interest in listening to the views and experience of *this person* will thus be of importance. Bengtsson (2005, p. 42) separates three thematics in phenomenological interviews: spontaneous and thus *unreflected experiences*, *reflected experiences* pointing to some form of self-understanding of something experienced, and finally, *perceptions of cognitive character* with different meanings. These thematics suggest that the texts generated through interviews may be of different kinds. For this reason, it is important to be aware of how a dialog is constructed and the fact that the researcher(s) also takes part in its co-construction and will thereby impact on what the interviewee shares in an interview. This motivates critical reflection on data credibility (K. Dahlberg et al., 2008).

There are a number of textbooks on research methods that deal with field work (see, e.g., Hammersley & Atkinson, 2019) applicable to studying social actions and thus opening up for what is taken as a given. Independently from the kind of data sources used and combined to facilitate credible analysis, it is important to distinguish first person experience from mediated (other people’s) experience.

### Sampling

Explicit sampling criteria are as central to an empirical phenomenological inquiry as to any research design. A requirement is that potential participants should have lived experience of the phenomenon inquired and are thus purposefully selected. Usually, phenomenological inquiries also have a combination of strategic and convenient sampling, as participants who fall within the inclusion criteria are asked to participate. Alternatively, consecutive sampling can be applied whereby participants are included in the order they turn up (e.g., admitted to a service). Homogeneity within the group of participants is desired in relation to some aspects, for example, mothers of children with hemophilia (see Myrin Westesson et al., 2013) whereas variation (strategic sampling) is often sought in relation to social background, living situation and gender, to name some aspects.

Deciding the number of study participants in advance is not a necessary given—what is important is to generate rich data, that is, data instilled with meaning that explore a range of experiences on the phenomenon studied. Thus, a small number of participants may be chosen for a case study analysis (e.g., Friberg & Öhlen, 2007). If so, additional participants may be recruited to generate more variety in the data, and these participants can be selected for meaning-rich data accordingly. Sometimes, additional participants are sampled or you choose to return to the same participants several times or to include different types of data, such as interviews, observations, diaries and blogs. The considerations and choices made must be described and motivated, like the decisions made at all stages of the inquiry process.

### Analysis

A common mode of operation in qualitative analyses is to move from a whole (e.g., all the data from all the participants) to parts (individual participants or situations in the field work; data parts or data segments) to a new whole (created by the final analysis, which becomes the result). In addition to richness of meaning, methodological transparency is a hallmark. We have described this above as related to the data generation phase, and here we will explore it in the analysis phase. In empirical phenomenological inquiry, a characteristic of the analysis is to be open to taking different directions in the analysis process and to accepting that final decisions might not be taken until later on. This is contrary to the principle of several other types of design, such as hypothesis-driven measurement, and of significance in empirical phenomenological inquiry due to the methodological requirement to do the phenomenon justice, as related to the complexity of the lifeworld.

The analysis is guided by openness. Hence, openness to the unexpected or even coming to an awareness of one’s own taken-for-granted assumptions (e.g., about what the study results may involve). K. Dahlberg et al. (2008) use the word “bridling” to hold back and avoid making hasty decisions, to avoid sticking rigidly to your own way of thinking and making a premature interpretation. In other words, it means being reflective throughout the process of inquiry. During the analysis work, this reflection is central when different data segments and meanings are to be put in relation to each other. Reflexivity is well described in qualitative research and can be related to considering both the researcher and the participant as bringing knowledge to the study. This is what Schutz (1997) calls a *stock of knowledge*, that is, previous experience (or experiential knowledge) thanks to the life we live. Thus, ideas and perspectives are culturally and socially shaped. As a researcher, it is important to consider what it means to be part of culture, tradition and history. Asking questions such as, *could it be different* or *what does this really mean from the perspective of the participant?* can be seen as a way of being alert to the preunderstanding during the analysis. Presenting and discussing preliminary analyses with others (colleagues, as well as participant stakeholder groups, such as patient representatives) can also facilitate reflection (e.g., in seminars).

Sometimes, the researcher is seeking the essence or core of the phenomenon, that is, the invariant of a phenomenon (see Primarily descriptive phenomenological methods, Table 1), and such an analysis process is described in Table 2. Sometimes, the researcher is primarily seeking to describe differences in meanings of a phenomenon due to various facets of experiences (see Primarily interpretive phenomenological methods, Table 1), and such an analysis process is described in Table 3.

### Reporting and Quality

Quality in phenomenological inquiry has great similarities with quality requirements in qualitative research in general. The debate has been intense concerning the pros and cons of replacing validity and reliability with more suitable concepts. Lincoln and Lincoln and Guba (1985) suggested trustworthiness as an alternative (including credibility, dependability, confirmability, transferability). Later, Morse (2015) proposes rigor and a return to the terms validity, reliability and generalizability, while K. Dahlberg and Dahlberg (2019) suggest objectivity, validity and generalizability. Generally, it can be said that the empirical phenomenological result discourse has genre features, as it clearly relates to and involves everyday language (which is not at all limited to phenomenological inquiry). However, variation could be sought here by putting everyday language and scholarly language side by side with more poetry-like meaning-rich texts. Some form of structure is nevertheless sought to facilitate reading and review.^5^

To point out quality aspects in phenomenological research, van Manen (2017b) talks about common misconceptions regarding phenomenological inquiry. These include the idea that all qualitative studies of experience are phenomenological. Instead, empirical phenomenological studies are to be characterized by phenomenological insight and preferably challenge the natural attitude and the taken-for-granted experience. Another misconception is that phenomenological data will automatically be generated in unstructured interviews. It is true, however, that an empirical phenomenological study must be planned in advance with an explicit knowledge interest so that the researcher can deliberately generate a certain type of data. Another idea is that empirical phenomenological results come from using certain steps in the research process. As shown in Tables 2 and 3, analytical steps are common.

Nevertheless, other researchers argue that an empirical phenomenological inquiry is about a way of thinking: formulating, developing and becoming familiar with and making use of a distinct perspective (i.e., ontological assumptions) rather than following some steps. Similarly, empirical phenomenological results do not consist of a list of themes. Rather, the results are built up as reflective texts that facilitate the reader to engage in meanings of human experiences and events and where themes *can* be used as a means to structure meanings disclosed in the analysis. Meanings can also be structured based on temporary or spatial aspects (Friberg & Öhlen, 2007) in narrative structures (Öhlén et al., 2013) and paradigm cases (Weiss, 2010). Zahavi (2019a) suggests that quality in empirical phenomenological research is especially shown in the results’ potential to inform change in practice.

## Criticism of Empirical Phenomenological Inquiry

Empirical phenomenological inquiry has been discussed by scholars both inside and outside the phenomenological community with varying intensity over the years. Critique from completely different paradigms might be characteristic of the latter part of the previous century. For example, Giorgi’s (2009) methodology was criticized by psychometrically oriented psychology researchers who proposed that the empirical descriptive phenomenology resulted in too uncertain and relativistic results. van Manen’s (1997, 2014) methodology has been criticized for lacking clear structure and being impressionistic. The debate from phenomenological scholars has been lively (Cf. For example, Giorgi, 2000, 2014; van Manen, 2018, 2019; Zahavi, 2019b) and accordingly important for the critical use of empirical phenomenological methodologies. Another critical comment is what Beck (2021) calls “method slurring” where parts from different methodologies are blended without underpinning arguments. Method slurring can also be linked to the broad tradition considering the study of *lived experience* as inherently phenomenological, which is not the case.^6^ Still an example is to assume “verbatim” transcripts from recordings of talk to correlate with the conversation hold in an interview and the participants’ “actual experience,” and not consider the perceptual act in sharing personal experience and the interpretive act in transcribing. If so, the phenomenological assumptions (e.g., an ambivalent synthesis between “life” and “world”) becomes transformed into claims of correlation. Furthermore, it examplify the challenges of applying ideas origninating in disciplinary fields external to a practice discipline like nursing (Lipscomb, 2022).

The complexity of using phenomenological philosophy in concrete empirical research has also been discussed by scholars external to the fields of phenomenological philosophy and empirical phenomenology, for example, Crotty (1996) and later by Paley (2017), and responded to by empirical phenomenologists (e.g., Giorgi, 2000; van Manen, 2017b, 2019). Above all, the risk proposed is that no use is made of the philosophical foundation. This is a severe and important critique since phenomenological philosophical foundation is claimed to be one (or *the* very) feature of empirical phenomenological inquiry. If this is *not* the case, it cannot simply qualify as phenomenological inquiry. This can be put into perspective in regard to other representatives claiming that empirical phenomenological knowledge contribution is invaluable, especially in terms of nuances and distinctions of meanings. In other words, it is important to take a position on and describe the philosophical foundations that could work as resources for study design and analysis and that are to be used throughout the inquiry (see, e.g., I. C. Berndtsson et al., 2007) but to leave out excess philosophy that is not used.^7^

## Concluding Reflections

Like all research, empirical phenomenological inquiry builds on a set of assumptions. However, what distinguishes it is explicating these assumptions and taking a foundation in phenomenological philosophy. Our intention, however, was not to analyze what may qualify fully and what might not. Rather, in relation to the aim of the paper, the intention is primarily to guide readers to the original sources of empirical phenomenological methodologies and phenomenological philosophy.

There are commonalities and differences in empirical phenomenological methodology (Beck, 2021) and the field is indeed motley and diverse. Our discussion of the descriptive and interpretive empirical phenomenological methodologies illustrates the main directions of this. Regardless of such differences, the crux of phenomenological inquiry is the focus on the experiences of *a phenomenon* and its analysis, along with critical reflection. Moreover, the generation of data emphasizes richness in meaning, as well as the focus in the analysis and in writing the report.

The rich tradition of empirical phenomenology inquiry in nursing has undergone a momentous revival since the millenium shift (Zahavi & Martiny, 2019). One interesting approach is technological advancement (implants, prostheses and the use of monitoring and self-care devices etc.) as an increasingly important element of health care, and the patient-practitioner relation (Forss & Ceci, 2017). Here, aspects such as embodiment can be understood in the intersection between philosophy, technology and nursing beyond the distinction of subject and object. It follows that postphenomenology (Ihde & Brook, 2003), and feminist phenomenology (Al-Saji, 2010; Zeiler & Käll, 2014) are proposed, as well as queer phenomenology (Heyes et al., 2016) whereby narrated experiences of gay persons against the background of history, gender and power are emphasized in data generation and analysis. These are examples of the continuation of the empirical phenomenological inquiry development. Since the millenium shift, the division between descriptive and interpretive methodologies is still noticeable, but there is little competitive tone in the debate; more of a scholarly debate (Burns & Peacock, 2019; Giorgi, 2014; Matua & Van Der Wal, 2015). Selected phenomenological concepts (e.g., lifeworld, intentionality, embodiment) and procedures in the process of analysis (e.g., distinguishing and labeling meaning units in longer text sequences) are often suggested in several empirical phenomenological methods (P. Ashworth, 2003; van Manen, 1997) and are still a significant indication that an inquiry is to be counted as phenomenological (Zahavi, 2019a).

## Notes

1. Methodology is used for principles and set of procedures for how to undertake empirical research (Cf. “methodology” in www.merriam-webster.com).

2. Examples include ethnography (Hammersley & Atkinson, 2019), narrative knowing (Polkinghorne, 1988) and Interpretive description (Thorne, 2016).

3. Husserl’s work is usually referred to as transcendental phenomenology and “the special method of the eidetic reduction by means of which the phenomena are described. Through the method of imaginative variation, (examples of instantiation and comparative examination) the invariant or eidetic aspects of a particular phenomenon are explicated” (Phenomenology online, 2022), and the invariant aspects transcend a phenomenon’s form of existence (experiential variations).

4. For qualitative research interviewing, see, for example, Dahlberg et al. (2008), Mishler (1986) and Riessman (2008).

5. Examples of how studies with various empirical phenomenological methodologies are concretized and reported, as well as what is to be considered quality in this research, are available in several edited volumes, which can provide suggestions for those who are novel to phenomenological inquiry. See, for example, Beck (2021), Bengtsson (2005), Berndtsson et al. (2019) and Dahlberg et al. (2019) and Chan et al. (2010).

6. Note, all inquiry into experience is not necessarily phenomenological. For example, the use of empirically inductive thematic- or content-oriented analyses are increasingly common, but not phenomenological in the sense presented in this paper. Nevertheless, arguments for such analyses are put forward and regarded as appropriate to generate knowledge about experience (e.g., Graneheim et al., 2017).

7. To learn more about how to apply and make use of the philosophical and methodological resources in empirical phenomenological inquiry, we recommend (as for all qualitative research methods) reading reports of where the method has been applied.
